# Improving Surgical Site Infection Prediction Using Machine Learning: Addressing Challenges of Highly Imbalanced Data

**DOI:** 10.3390/diagnostics15040501

**Published:** 2025-02-19

**Authors:** Salha Al-Ahmari, Farrukh Nadeem

**Affiliations:** 1 Department of Information Systems, Faculty of Computing and Information Technology, King Abdulaziz University, Jeddah 21589, Saudi Arabia; 2 Department of Computer and Information Systems, Applied College, King Khalid University, Abha 61421, Saudi Arabia

**Keywords:** grid search cross-validation, imbalanced classification, machine learning algorithm, oversampling, surgical site infection, undersampling

## Abstract

**Background**: Surgical site infections (SSIs) lead to higher hospital readmission rates and healthcare costs, representing a significant global healthcare burden. Machine learning (ML) has demonstrated potential in predicting SSIs; however, the challenge of addressing imbalanced class ratios remains. **Objectives**: The aim of this study is to evaluate and enhance the predictive capabilities of machine learning models for SSIs by assessing the effects of feature selection, resampling techniques, and hyperparameter optimization. **Methods**: Using routine SSI surveillance data from multiple hospitals in Saudi Arabia, we analyzed a dataset of 64,793 surgical patients, of whom 1632 developed SSI. Seven machine learning algorithms were created and tested: Decision Tree (DT), Gaussian Naive Bayes (GNB), Support Vector Machine (SVM), Logistic Regression (LR), Random Forest (RF), Stochastic Gradient Boosting (SGB), and K-Nearest Neighbors (KNN). We also improved several resampling strategies, such as undersampling and oversampling. Grid search five-fold cross-validation was employed for comprehensive hyperparameter optimization, in conjunction with balanced sampling techniques. Features were selected using a filter method based on their relationships with the target variable. **Results**: Our findings revealed that RF achieves the highest performance, with an MCC of 0.72. The synthetic minority oversampling technique (SMOTE) is the best-performing resampling technique, consistently enhancing the performance of most machine learning models, except for LR and GNB. LR struggles with class imbalance due to its linear assumptions and bias toward the majority class, while GNB’s reliance on feature independence and Gaussian distribution make it unreliable for under-represented minority classes. For computational efficiency, the Instance Hardness Threshold (IHT) offers a viable alternative undersampling technique, though it may compromise performance to some extent. **Conclusions**: This study underscores the potential of ML models as effective tools for assessing SSI risk, warranting further clinical exploration to improve patient outcomes. By employing advanced ML techniques and robust validation methods, these models demonstrate promising accuracy and reliability in predicting SSI events, even in the face of significant class imbalances. In addition, using MCC in this study ensures a more reliable and robust evaluation of the model’s predictive performance, particularly in the presence of an imbalanced dataset, where other metrics may fail to provide an accurate evaluation.

## 1. Introduction

SSIs can lead to septic complications, resulting in increased hospitalization stays and costs and even death [1,2]. They can occur within 30 days after surgery or up to 90 days after a prosthetic implantation [3]. However, most SSIs are preventable by standard protocols [4]. A frequent form of hospital-acquired infection (HAI), SSIs affect approximately 30% of patients undergoing surgery in developing countries [1,2,4,5]. Accounting for approximately 20% of all HAIs, Saudi Arabiaranks second among high-income countries [6]. Up to 5% of approximately 27 million surgeries in the United States are associated with SSIs, which can manifest between 1 and 30 days following certain procedures [7]. Advanced SSI prediction can significantly lower SSI instances, benefiting patients and healthcare organizations [8].

SSI prediction is a generic problem based on a variety of symptoms and criteria, considering infections observed in many categories. Most previous studies [2,7,8,9] have discussed the implications of predictive SSI models by manually classifying patients according to critical factors including the existence of comorbidities, risk level, and contamination risk, as well as features of the superficial wound. Some studies have also shown that the AUC of similar ML-based prediction models can reach up to 89% [10,11]. Nonetheless, it is crucial to note that the possibility of a low positive predictive value persists, indicating restricted accuracy in identifying actual positive cases. This discrepancy often arises when the model exhibits bias towards the majority class, particularly in imbalanced datasets. Such bias can mask the model’s true performance, leading to a phenomenon known as the accuracy paradox [12].

Elevated accuracy metrics do not inherently indicate a superior model, as the model may exhibit bias towards the majority class (No-SSI), thereby obscuring the outcomes [12]. Further research is required to develop and validate ML algorithms for SSI prediction, especially given the difficulties presented by severe class imbalance in the national dataset. In this regard, the present study improved the prediction performance by tuning ML models with hyperparameters and adjusting different undersampling and oversampling strategies to handle an imbalanced classification problem. The oversampling and undersampling methods each have their own benefits and drawbacks [12,13]. Oversampling can effectively boost the number of instances in the minority class, thereby mitigating bias in favor of the majority class. Oversampling can facilitate the model’s learning of minority-class patterns and characteristics by increasing the availability of instances in this class, thereby enhancing its capacity to generalize to novel instances. This can also result in overfitting and the creation of artificial samples that do not accurately represent the minority class. Most class instances can be effectively reduced through undersampling, thereby concentrating on the most informative instances. Undersampling can help to concentrate on the most informative instances and decrease the noise and redundancy in the dataset. Conversely, this approach can also result in information loss, causing the removal of instances crucial to the model’s performance. It is essential to use oversampling and undersampling methods with careful consideration, along with other preprocessing techniques, such as feature selection and normalization [14]. The selection of an oversampling or undersampling method should be determined by the unique features of the dataset and the objectives of the machine learning model [12]. This study investigates the impact of various multiple oversampling and undersampling techniques on the prediction of SSIs for diverse machine learning models. The SSI dataset we selected exhibits highly subpar prediction performance for most minority classes when no oversampling or undersampling technique is applied. In several aspects, we believe that this study contributes to the literature concerning the handling of highly imbalanced classification in real-world scenarios. The main contributions of the paper are summarized as follows:

1. Comprehensive Model Evaluation: An extensive comparison of seven ML models across different oversampling and undersampling techniques was conducted, providing a holistic view of model performance in SSI prediction.
2. Grid search cross-validation (CV) was employed for in-depth hyperparameter tuning, aiming to optimize model performance while minimizing overfitting. This method involves a systematic exploration of various parameter combinations and ensures a comprehensive evaluation by using different data subsets for training and validation in each fold. Such a strategy enhances the generalizability and reliability of the model, providing a robust and unbiased assessment crucial in critical fields such as healthcare.
3. Efficient filter selection methods were employed as initial steps in handling high dimensional, imbalanced datasets, thereby offering a computationally cost-effective feature selection approach.
4. An adaptive decision threshold strategy was implemented to achieve a consistent recall of 70% across models, effectively addressing the issue of imbalanced classes and improving the predictive accuracy for the minority class in SSI detection.

These contributions underscore the significance of our research in developing a robust model for SSI prediction, particularly in addressing the challenges of imbalanced data to reflect real-world scenarios in the healthcare domain. This paper is organized in the following manner: Section 2 explores the related literature. Section 3 outlines the concept of an imbalanced classification problem and discusses various balancing techniques. Section 4 describes the methodology used to train and evaluate predictive models. Section 5 introduces the results and findings. Section 6 summarizes the limitations of this study and its future implications. Section 7 concludes the research.

## 2. Related Work

Bartz-Kurycki et al. [15] used the National Quality Improvement Program database by the American College of Surgeons (AQS-QIP), specifically pediatric data from 2012 to 2015, and applied different ML models to maximize accuracy. They examined four prediction models: one based on RF and three based on LR. To identify crucial predictors, the researchers combined insights from previous literature and clinical expertise with data-driven feature selection techniques, aiming to optimize model performance and ensure clinical relevance. The best results were acquired from LR combined with the important features generated by RF, with a 69% area under the receiver operating characteristic (AUC). Over 10 years of surveillance, Song et al. [16] acquired data from a significant research health center in New York, including information on patients with paramount pacemakers, and applied three classification models—LR, DT, and SVM. Of them, LR achieved the best performance (72.9%); however, the model exhibited restricted clinical applicability due to its low predictability (i.e., PPV or precision rate = 32%).

Chen et al. [17] compared GB, LR, RF, and artificial neural network (ANN) classifiers to determine superficial and deep/organ-space SSIs by applying ML algorithms, including ANN, RF, and LR. The findings showed that ANN, with a 0.769 (76.9%) AUC, showed the best performance in detecting SSIs. The most suitable SSI prediction technique was evaluated in patients who underwent collateral surgery using data from the AQS-QIP database from 2012 to 2019. The dataset includes 275,152 patients from all samples: only 10.7% experienced SSIs. Performance evaluation of the selected prediction models was performed using the AUC metric, and ANN demonstrated the best performance (76%).

Al Mamlook et al. [7] developed and validated ML approaches to improve the prediction of SSI occurrence. Using National Surgical Quality Improvement Program data, the performance of several ML approaches was compared based on precision, accuracy, and AUC. A total of 2,882,526 surgical procedures were identified for the creation of a predictive SSI model. The findings indicated that the deep neural network (DNN) achieved the best predictive performance (precision = 0.85, accuracy = 0.85, and AUC = 85%), surpassing the other techniques by 10-fold, including tree-based DT, RF, simple LR, kernel SVM, and ANN. However, there was a significant imbalance between the two classes, with SSI cases representing only 0.32% of the entire dataset.

Scala et al. [18] analyzed surgical data from a single center, and ML models, including RF, GB, and XGBoost, were compared. The XGBoost model achieved moderate performance, with an AUC of 0.69, precision of 0.3, and sensitivity of 0.83. The study highlighted the challenges associated with imbalanced datasets, which reduced the models’ predictive accuracy.

Petrosyan et al. [10] routinely collected administrative data of three different institutes from a National Surgical Quality Improvement Program database in Canada. RF was applied using a stepwise feature selection method and validated using a 10-fold cross-validation to improve accuracy. The results showed that RF achieved a high level of performance, with an AUC of 89.43%, but was limited in other metrics such as precision (34%), in addition to exhibiting a high level of imbalance between the two classes, with SSI cases representing 5% of the entire dataset (14,351 patients).

Similarly, Wu et al. [19] conducted a single-center study of total hip and knee arthroplasties, employing XGBoost to predict complex SSIs using administrative data. The best XGBoost model obtained an AUC of 0.90, confirming its robust predictive ability. This study highlighted challenges, including the potential discordance in SSI incidence rates based on follow-up days, imbalances in data affecting model performance, and reliance on the quality of clinical documentation.

Xiong et al. [11] reviewed patients who underwent lumbar interbody fusion from 2019 to 2021 to implement and validate different ML models for the prediction of SSIs and to explore whether using the class-balancing method, i.e., SMOTE, is effective for enhancing the quality of prediction and performance. Preliminary analysis showed that the performance of the AdaBoost classifier was 87%. However, the precision rate, or precision, was only 23%, and the small sample size may have led to overfitting. These studies are summarized in Table 1.

Studies using national databases have revealed two interesting points. First, the results of previous studies that compared ML models indicated that the best performance was achieved using the RF and AdaBoost classifiers [10,11], with a AUC values of 89% and 87%, respectively. However, they have limited clinical implications due to their low recall (23% and 34%, respectively). Second, sampling methods such as SMOTE were applied to balance the dataset [16]. However, the single-center, retrospective nature of this study [11] might have created bias in selection, restricting its generalizability, while other studies did not address imbalanced classification problems that affect prediction model performance. The number of features generated from national databases is typically high, but not all are relevant. Therefore, the feature selection step should ensure the inclusion of key features from the original feature sets discussed in recent studies. For instance, Petrosyan et al. [10] employed stepwise feature selection before applying the RF model. Other studies, such as [7,11,16,17], did not use feature selection methods, except one [15], which used previous literature and clinical experts to feed the models with only a limited set of important features.

Additionally, several significant metrics were computed in the test set to compare the results with those of other studies, including accuracy, recall (sensitivity), precision (PPV), and F1 score [7,11,15,16,17]. The results from previous studies [7,10,11] showed high to moderate AUC rates; however, a low positive predictive value of 34% [10] indicates limited accuracy in identifying true-positive cases, highlighting the potential shortcomings of relying solely on accuracy metrics. Despite a high overall accuracy, a model may not necessarily be of high quality or clinically useful. Previous studies have reported high to moderate Area Under the Curve (AUC) rates [7,10,11], suggesting promising overall model performance. However, a low positive prediction rate reveals limitations in these models. For instance, one study reported a low positive predictive value of 34%, indicating limited accuracy in identifying true-positive cases [10].

This discrepancy often arises when the model exhibits bias towards the majority class, particularly in imbalanced datasets. Such bias can mask the model’s true performance, leading to a phenomenon known as the accuracy paradox [12]. Thus, a gap was observed in past studies related to resolving the issues of imbalanced classification when evaluating predictive ML models. The effect of the accuracy paradox on models in which high accuracy does not reflect high predictability on imbalanced datasets is particularly concerning.

## 3. Imbalanced Classification Problem

**Figure 1:**
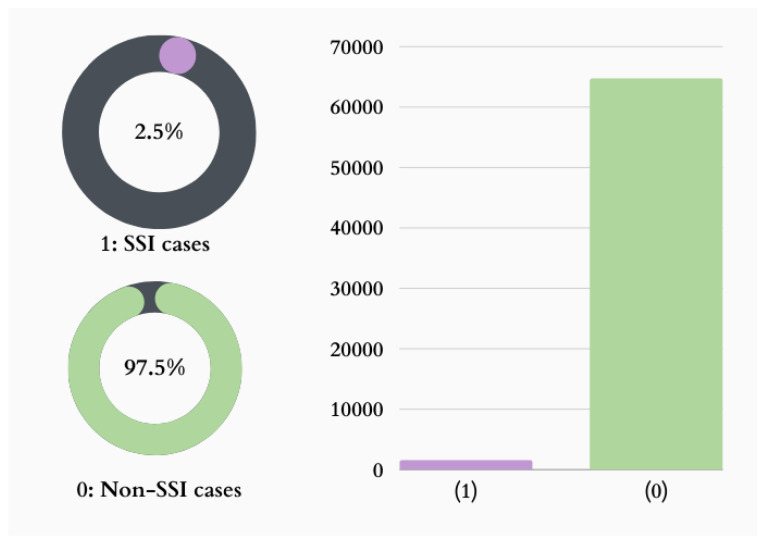
Data distribution in the two classes.

According to some experts, an unbalanced classification problem can negatively affect the effectiveness of common data mining approaches [11,20,21,22,23]. Datasets become more complicated and unbalanced as memory and computer capacity increase rapidly. This is particularly severe in the setting of clinical data because several cases in the majority class might have been caused by a single unusual occurrence [20]. Predictive modeling for classification problems in ML is tasked with predicting the class a given observation belongs to. However, the distribution of samples across various classes is frequently skewed (Figure 1). Unbalanced classification has been found to have worse prediction accuracy, particularly for the minority class, which presents a barrier in many real-world ML applications [21,22]. To achieve more precise outcomes and avoid the “accuracy paradox”, it is crucial to ensure that a high accuracy value aligns with a high-quality model. This is because a model that is biased toward the majority class can mask the insights gained from the data [23]. Thus, it is crucial to employ techniques that minimize this phenomenon when the imbalance problem arises.

In the current research, specialized approaches for adjusting these data imbalances were frequently used. The proposed method is based on a real healthcare-domain dataset that can significantly explain an imbalanced classification problem. Here, we predict whether a patient will develop postoperative SSI based on the given features. Figure 1 explains the class distribution in our dataset: 0 means the patient has no SSI, and 1 means the patient has an SSI. Only 2.7% of patients presented with an SSI. Therefore, the SSI rate in our dataset is a classic imbalanced classification problem.

To obtain a balanced distribution between the two classes, sampling procedures (also referred to as external techniques) employ a preprocessing stage at the data level. The imbalance ratio in the training data was reduced using either undersampling, oversampling, or both [21]. Undersampling eliminates examples of the dominant class unless a more even distribution is attained [24]. The most basic undersampling technique, known as random undersampling, involves randomly eliminating occurrences from the training dataset’s majority class. The oversampling strategy involves copying and repeating the instances of minority classes in the initial dataset until a more even distribution is obtained [21]. However, random undersampling and oversampling techniques provide good results [14]. The proposed SMOTE approach offers several improvements that make it more selective for synthesized instances in the majority class [25]. The most common addition to SMOTE is adaptive synthetic (ADASYN) sampling, which creates synthetic samples that are inversely correlated with the proportion of instances in the minority class [12].

## 4. Method

**Figure 2:**
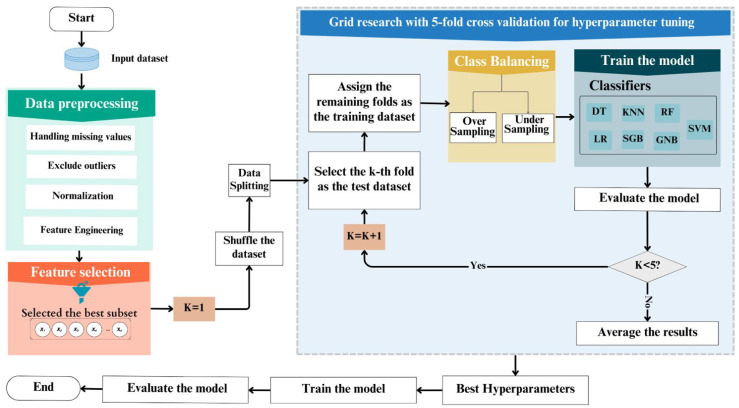
Flow diagram of the research methodology.

This study aimed to predict SSI occurrence following different surgical procedures and to determine if balancing and feature selection techniques could improve model predictability, with high performance for different evaluation metrics. A flowchart for SSI prediction using the ML models, namely LR, GNB, KNN, DT, RF, SGB, and SVM, is presented in Figure 2. Specifically, the dataset was preprocessed. After that, a grid search with 5-fold cross-validation tuned the model hyperparameters with the use of different sampling techniques, including oversampling techniques (random, SMOTE, and ADASYN) and undersampling techniques (random, cluster centroids, and instance hardness threshold). The dataset was divided into 5 subsets, with each subset used once as the validation data while the model was trained on the remaining 4 subsets. This process was repeated 5 times, with each subset serving as the validation data exactly once. The average performance across all folds was then computed to evaluate the model’s performance under different hyperparameter configurations. Finally, with the best hyperparameters obtained, the prediction model was trained and evaluated. This study was approved by the Central Institutional Review Board (IRB) committee of the General Directorate of Research and Studies at the Ministry of Health (MOH), Kingdom of Saudi Arabia (IRB Log Number: 23-86-E). Figure 2 shows a diagram of the research methodology.

### 4.1. Dataset

The dataset for this study was obtained from the General Administration for Infection Control in Health Facilities at the MOH, Kingdom of Saudi Arabia. It contains SSI surveillance data collected between January 2017 and December 2021 from 109 Saudi Arabian MOH facilities registered with the Health Electronic Surveillance Network (HESN). As part of the requirement mandated by the MOH, all patients who developed an SSI 30 or 90 days post surgery were documented and reported. The data were entered into the HESN program by the staff of the participating hospitals. The dataset includes data of pediatric and adult patients who underwent surgery and was derived from four different types of hospitals: general hospitals, women and children’s hospitals, pediatric hospitals, and cardiac centers.

### 4.2. Data Preprocessing

The dataset comprises 65,514 patient records with 80 distinct features. However, some of the features were irrelevant to the study because they represent hospital information, yielding no significant parameters in this scenario; therefore, these columns were omitted. Missing data were handled by either dropping features with ≥80% missing values or imputing values for features with <80% missing data. Outliers are data points far below or above the typical values contained in a dataset. They can make ML models biased if not detected and handled effectively. Outliers are usually different among the data points; for instance, height, weight, and body mass index outliers may include some unusual values. The remaining records totaled 64,793, with 1632 SSI cases, and the dataset was pre-defined into two groups based on the SSI and non-SSI status reported in the dataset, where the binary target variable was defined as “Yes” (SSI) or “No” (non-SSI). The categorical variable was encoded with one-hot encoding, and the numerical variables were normalized. We divided the dataset into training and test sets in a 70:30 ratio. We utilized the stratified strategy instead of random splitting to keep the class proportions in the training and test sets consistent with those in the original dataset.

### 4.3. Statistical Analysis

We used Python (version 3.9.12) and its scientific computing libraries for statistical and ML analysis. We employed the chi-square test to examine the relationship between the presence of an SSI and categorical variables. Table A2 and Table A3 in Appendix A present the distributions of various factors by SSI occurrence, with data represented as frequencies (N) and percentages (%). These tables also include *p*-values, indicating the statistical significance of associations between factors and SSI occurrence. We conducted a normality test for numerical variables to guide the selection of statistical methods. Based on the results of the normality test, we employed the Mann–Whitney test, also known as the independent *t*-test, to examine associations between numerical variables and SSI occurrence, as presented in Table A1 in Appendix A. *p*-values less than 0.05 were considered statistically significant.

### 4.4. Filter Feature Selection

In high-dimensional datasets, feature selection is crucial for improving model performance and reducing computing complexity [26]. Filter methods are highly regarded for their effectiveness in managing large datasets [27]. They differ from wrapper and embedded methods in that they assess the importance of features based solely on their inherent properties rather than relying on learning algorithms [26]. This feature allows filter methods to be less computationally intensive and scalable to large datasets, making them well-suited for initial feature selection on large datasets.

We used the Chi-square test to filter feature selection and evaluate the relationship between categorical features and SSIs occurrence. Features with a higher chi-square score and *p*-values less than 0.05 are considered more relevant because they demonstrate a stronger association with the target variable. The test also evaluated the difference between the observed value ($O_{i}$) and expected value ($E_{i}$). The chi-square test was calculated using Formula (1):

(1) $$X^{2}=\sum_{i=1}^{n}\frac{{\left(O_{i}-E_{i}\right)}^{2}}{E_{i}}$$

**Figure 3:**
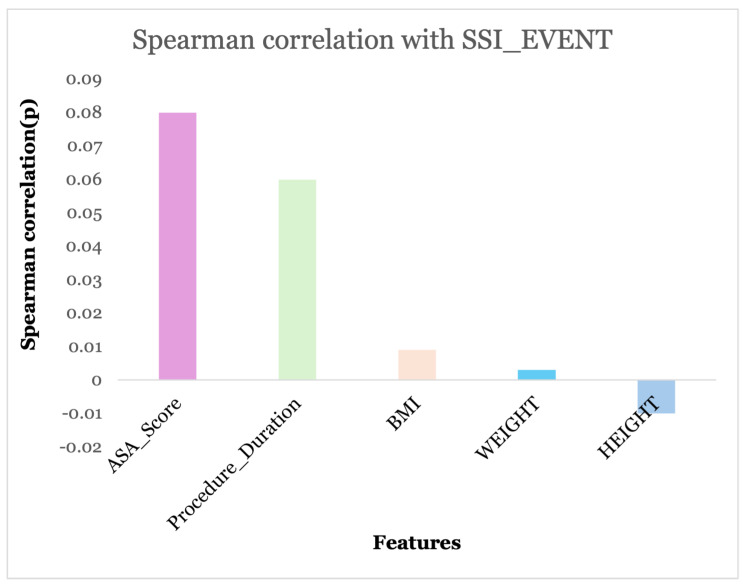
Numerical features selected based on Spearman correlation.

Conversely, as illustrated in Figure 3, Spearman correlation was employed to filter features and assess the relationship between numerical attributes and the target attribute (SSI_EVENT). Correlation values of 0 signify a weak or no link with SSI_EVENT, whereas numbers approaching +1 or −1 denote significant positive or negative monotonic associations, respectively. ASA_SCORE and PROCEDURE_DURATION exhibit minimal positive correlations with SSI_EVENT; however, they may retain relevance, particularly within medical data, where even slight correlations can be meaningful in prediction models.

**Figure 4:**
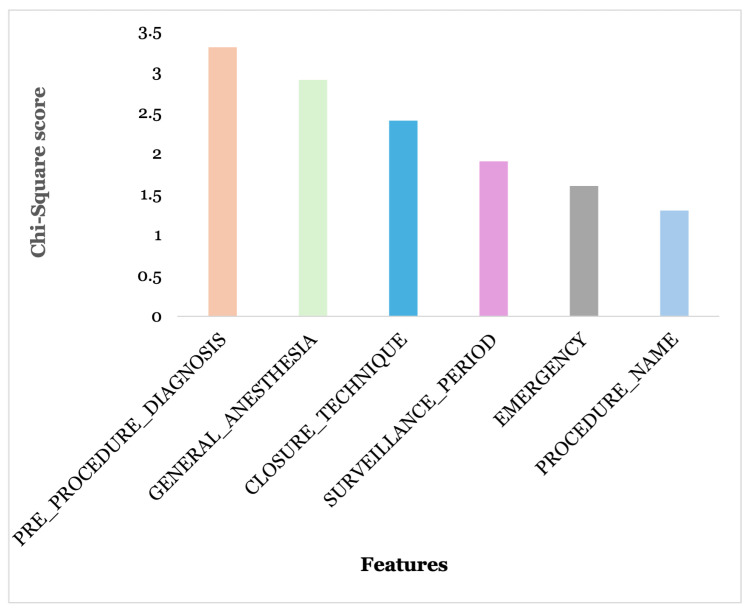
Categorial features selected based on chi-square test.

Based on the chi-square scores and *p*-values for the categorical features, we retained only the features most relevant to SSI_EVENT. All excluded features had *p*-values greater than 0.05 and very low chi-Square scores, as shown in Figure 4.

Ultimately, the selected features were procedure name, surveillance period, emergency, pre-procedure diagnosis, closure technique, general anesthesia, procedure duration, and ASA score.

### 4.5. Class Balancing

Sampling is used to handle an imbalanced dataset, and oversampling and undersampling are the two most common sampling techniques [24]. Here, each method was used with a different sampling strategy to control the distribution between the majority and minority classes in the training set; for example, the optimal sampling strategy was used for SMOTE oversampling, which resulted in a sampling strategy of 0.2, with k_neighbors set to 7.

#### 4.5.1. Oversampling

Oversampling is a data analysis technique used to adjust the class distribution of a dataset [25]. In this study, different duplication methods for the minority class were used to balance the two distributions. The simplest approach is the random oversampling technique, where random points from the minority class are selected and duplicated to increase their number [28]. SMOTE is a form of oversampling used to select the examples nearest in the feature space to create new synthetic examples and draw a line between examples [29].

ADASYN is another technique that employs a weighted distribution to generate synthetic data for various minority-class examples, considering their multiple levels of learning difficulty [28]. It prioritizes the production of more synthetic data for difficult-to-learn minority-class instances than simpler-to-learn minority samples [30].

#### 4.5.2. Undersampling

A range of undersampling methods is employed to adjust the class distribution in a classification dataset with an imbalanced class distribution [24]. An unbalanced class distribution comprises one or more minority classes with limited instances—and vice versa for majority classes with several instances [31]. Here, we employed various undersampling strategies, including the cluster centroid (CC), random, and instance hardness threshold (IHT) methods.

The basic undersampling method involves randomly choosing examples from the majority class and removing them from the training dataset, also known as random sampling [24]. The expansion of random undersampling is more discriminative when samples are deleted from the majority class [31].

CCs are significant in balancing class distribution by creating synthetic data for the majority class based on the centroids of clusters formed by the minority class [32]. In this method, the majority class is minimized by selecting a subset of majority-class examples representing clusters formed by the minority class. This subset is determined by identifying centroids of the minority-class clusters. Synthetic data are then generated by creating new examples close to these centroids [32].

On the other hand, the IHT method selectively undersamples the “hard” majority class instances based on difficulty classification. A classifier is trained, and instances with high uncertainty or low predicted probability of belonging to the majority class are considered hard [33]. Instances that exceed a set threshold are undersampled, which reduces dataset imbalance and potentially improves classification performance [33]. The majority class is reduced by providing a constant check and comparison between the minority class and whatever was left to examine the process of obtaining the best distribution of the majority class. This process is iterated, even when some information is lost.

### 4.6. Classifier Training

Seven ML classifiers, including LR, SVM, DT, GNB, KNN, RF, and Stochastic GB (SGB), were developed and tested for SSI prediction, using Python package Scikit-learn version 3.9.12 [34,35].

#### 4.6.1. LR

The proposed method employs a supervised ML algorithm to handle classification problems [15]. LR is adequately applied when there are categorical values in the target class that can be used to categorize and predict the disease. The statistical method is appropriate for analyzing the relationship between dichotomous or binary outcomes (0/1) using a set of independent predictors.

#### 4.6.2. DT

Instances are categorized according to feature values. Notably, in a DT, each node indicates a feature in an instance that must be categorized, and each branch provides a possible value for the node [16]. The best-set variable is attributed to the root dividing the algorithm into parts to unmix the dataset. Iterative splitting occurs until the data are grouped into homogenous partitions.

#### 4.6.3. KNN

KNN is another well-known classification method because of its easy interpretation and quick computation [35]. It works well with separate target classes by calculating the distance of the point of a query for each instance to find the K minimum distances. The KNN algorithm is an effortless algorithm in which data are grouped into coherent subsets or clusters, which categorize the data according to their similarity relative to the trained dataset, assigning the input variable to the nearest neighbors.

#### 4.6.4. SVM

SVM, a type of supervised ML approach, aims to find an n-dimensional repeatable hyperplane that maximizes the distance between the support vectors of two different class labels [36]. It is a classification–regression method developed for multiple classification problems, applied simultaneously with feature selection.

#### 4.6.5. GNB

This classification method assigns a label to a class, increasing the posterior probability of the individual sample. This assumption applies to voxel contributions that are conditionally independent, following Gaussian distribution rules. The decision rule of GNB obeys a discriminant function in each class [16]. GNB assumes that each variable predicts the outcome attribute independently, such that the final prediction categorizes the dependent variable into each group based on the highest probability of the respective set of items [32].

#### 4.6.6. SGB

SGB is applicable when the model has to perform classification or regression. Generally, GB constructs an ensemble of weak DTs as a stage-wise model to minimize a differentiable loss function [35]. The SGB approach, which proceeds when the model’s loss function is negative, avoids overfitting by randomly generating training subsets from a much smaller percentage of the training data at each stage of the training process [34].

#### 4.6.7. RF

A group of classifiers that use trees to classify data constitutes the RF ensemble approach [15]. The proposed RF constructs many DTs, and each tree performs independent categorization. The RF selects the class with the highest number of votes, guaranteeing each DT’s independence to realize better generalizability and classification accuracy.

### 4.7. Hyperparameter Optimization

In our study, we implemented grid search with 5-fold cross validation to tune hyperparameters for multiple ML models, ensuring each model was optimally suited for handling our imbalanced datasets. We utilized both oversampling and undersampling techniques to create balanced datasets, a crucial step in addressing class imbalance that often skews model performance. Each fold in the dataset served exactly once as a test set, while the remaining folds were used for training, allowing us to effectively evaluate and refine the hyperparameter settings for each model across various data subsets. We defined and cross-validated hyperparameters for each algorithm, providing a structured approach to minimize overfitting risks and accurately estimate model performance. This methodical configuration enabled us to pinpoint the hyperparameter set that best accommodated each model, optimizing for performance metrics that were particularly critical, given the present class imbalances. The process of hyperparameter selection and model training was thoroughly documented, with the results and optimal settings summarized in Table 2. Additionally, we evaluated each optimized model against different sampling methods to determine the most effective approach for balancing our datasets. This benchmarking was vital to enhance the reliability of our proposed machine learning pipeline. By adjusting classification thresholds, we managed to achieve up to a 70% recall rate, which significantly reduced biases toward the dominant class. This comprehensive analysis ensured that we selected the best-performing models, hyperparameters, and data-balancing techniques, culminating in a robust framework ready for deployment or further investigation.

### 4.8. Model Evaluation

**Figure 5:**
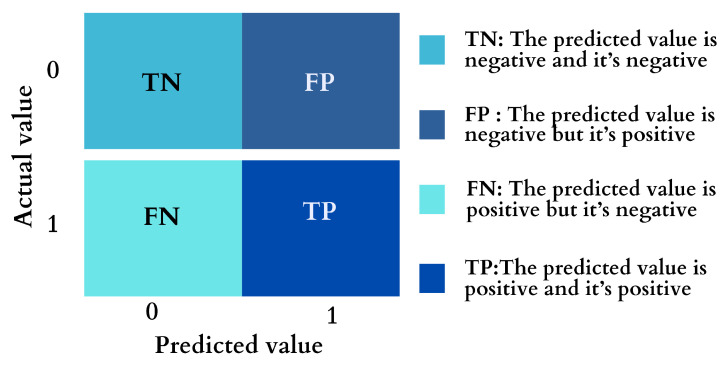
Confusion matrix.

A confusion matrix was applied to assess the performance of the prediction model, considering appropriate measures of performance for an imbalanced dataset [22]. As shown in Figure 5, it includes four terms: true positive (TP), where the occurrence of the instance is correctly classified as Yes or 1; false positive (FP), where the presence of the instance is incorrectly classified as Yes or 1; true negative (TN), where the presence of the instance is correctly classified as No or 0; and false negative (FN), where the occurrence of the instance is incorrectly labeled as No or 0. Based on the confusion matrix, the first performance metric is accuracy, which represents the overall predictive ability of the developed ML model.

Another performance metric is the AUC, a performance measurement metric for ML algorithms in classification problems.

The ROC curve is a probability curve that plots the sensitivity (i.e., TP rate) on the vertical axis and the specificity (i.e., TN rate) on the horizontal axis, computing the critical AUC. The AUC lies between 0.5 (worst predictive ability) and 1 (best predictive ability) [37].

Differing from previous studies, this study used the precision–recall plot (PR_AUC), an important evaluation tool for binary classification models, especially when dealing with imbalanced datasets [38]. Various important metrics were also calculated in the test set to compare the results with those reported in the literature, such as accuracy (Acc) (2), precision (3), recall (4), and F score (5), as follows:

**Accuracy**—While this is the most common parameter, it is not reliable. Assuming a dataset with imbalanced classes, the accuracy score will be high, but because its performance is based on the class with the most data points, the model will be biased, although with a high accuracy score [22].

(2) $$\mathrm{Acc}=\frac{\left(\mathrm{TP})+(\mathrm{TN}\right)}{\left(\mathrm{TP})+(\mathrm{TN})+(\mathrm{FP})+(\mathrm{FN}\right)}$$

**Precision**—The proportion of cases that belong to the positive class is expressed as precision [22].

(3) $$\mathrm{Precision}=\frac{\mathrm{TP}}{\left(\mathrm{TP})+(\mathrm{FP}\right)}$$

**Recall**—The TP and recall rates are the same. The recall calculation is the same as the sensitivity calculation and summarizes how well the positive class was anticipated [22]. We used threshold adjustment to achieve the desired recall level. This technique is particularly useful when prioritizing recall over precision in a classification mode [39]. As in SSI prediction scenarios and other medical diagnoses, FNs are more costly than FPs.

(4) $$\mathrm{Recall}=\frac{\mathrm{TP}}{\left(\mathrm{TP})+(\mathrm{FN}\right)}$$

**F1 Score**—The F1 score is the harmonic mean of recall and accuracy, with scores ranging between 0 and 1 [22].

(5) $$F1\;\mathrm{Score}=2*\left(\frac{\mathrm{Precision}*\mathrm{Recall}}{\mathrm{Precision}+\mathrm{Recall}}\right)$$

Recently, a different metric that considers all components of the confusion matrix was introduced in [40]; it is suitable for imbalanced data. The Matthews Correlation Coefficient (MCC) [41] is defined as the geometric mean of the regression coefficients of a problem and its dual. On the one hand, MCC = 1 indicates that both classes are satisfied perfectly, as observed in the alternative metrics. In contrast, an MCC of −1 shows complete disagreement between the actual and predicted classes. An MCC of 0 indicates a random prediction.

(6) $$\mathrm{MCC}=\frac{\mathrm{TP}\cdot \mathrm{TN}-\mathrm{FP}\cdot \mathrm{FN}}{\sqrt{\left(\mathrm{TP}+\mathrm{FP})(\mathrm{TP}+\mathrm{FN})(\mathrm{TN}+\mathrm{FP})(\mathrm{TN}+\mathrm{FN}\right)}}$$

## 5. Results

### 5.1. Approach A: Comparison of Results Using Oversampling Techniques

Table 3 represents the comparative analysis of the different ML models after applying oversampling methods, such as simple random sampling, SMOTE, and ADASYN. The measures assessed were accuracy, precision, recall, F1 score, AUC, and PR_AUC, with the greatest focus being on the minority class (label “1”).

The ML models for SSI prediction demonstrated a distinct performance ranking. Among the oversampling techniques, RF with SMOTE produced the highest precision (91%) and F1 score (79%). These models, including the RF variants with random oversampling and ADASYN, exhibited superior predictive power while maintaining a 70% recall rate.

Ensemble methods such as RF and SGB effectively capture complex, nonlinear relationships in the high-dimensional feature space of SSI risk factors [42]. These algorithms surpass traditional methods, yielding F1 scores ranging from 66 to 79%. Ensemble methods excel at discovering intricate patterns and relationships in high-dimensional feature spaces. The combination of their CV techniques enhances their resistance to overfitting and results in superior generalization capabilities.

The effectiveness of the oversampling techniques differed among the algorithms. SMOTE generated optimal results for RF and KNN models. Therefore, accurate selection of the sampling technique is important, as it aids in effectively handling class imbalance. SMOTE’s success stems from its ability to generate authentic examples that accurately depict the distribution of the minority class, thereby enhancing the training set’s balance and insight. Recall for all models reached the 70% mark; however, precision demonstrated notable differences.

The RF model demonstrated significantly higher precision (85–91%) than the LR and GNB models (6–11%). The significant disparity in SSI prediction between sensitivity and specificity highlights a substantial challenge that must be addressed. The poor efficiency of the LR and GNB models may be attributed to their fundamental assumptions on data linearity and independence, which are frequently contravened in complicated medical datasets. Adjusting decision thresholds for a consistent 70% recall effectively prioritizes the identification of potential SSI cases. This method agrees with the clinical need for early detection, with prioritization over higher precision in some models.

A more nuanced understanding of model performance can be realized by evaluating beyond simple accuracy using metrics such as F1 scores and confusion matrices. This method overcomes the “accuracy paradox” issue observed in imbalanced SSI prediction datasets, delivering reliable evaluations of each model’s predictive abilities.

Overall assessments with measures other than accuracy—mainly F1 scores and confusion matrices—shed more light on the models’ performances. This approach neutralizes the accuracy paradox common in imbalanced datasets; the efficacy of each model in terms of SSI prediction is determined in this manner.

#### 5.1.1. Confusion Matrix

An essential role was assigned to the concept of the confusion matrix to handle and alleviate accuracy paradox issues, with explicit reference to SSI prediction based on clinical data relevant to patient outcomes. The accuracy paradox results when there is an increase in the distance between datasets. This means that while a model can provide high accuracy, it can also poorly forecast the minority class, which, in medical diagnoses, is more valuable than the majority class. The confusion matrix supports going beyond general accuracy and provides a more insightful analysis of TP, TN, FP, and FN in the context of class imbalances. When the dataset is highly imbalanced, with few positive cases, the model achieves high accuracy by predicting the majority class. A model that predicts all cases as non-SSI would have a 95% accuracy rate if 95% of the cases were, indeed, non-SSI. Likewise, a model’s inaccuracy in identifying SSI cases could have disastrous consequences in a medical context. Hence, the confusion matrix directly addresses this issue by dividing the types of errors that the model makes:

- **FPs:** Misclassifying non-SSI as SSI cases may lead to unnecessary interventions. However, FPs are less severe than FNs.
- **FNs:** Failing to identify actual SSI cases is significantly dangerous because untreated infections can worsen over time, leading to critical consequences.

In this study, the oversampling technique led to significant differences in FP and FN rates among the six ML models according to their confusion matrices. Due to these differences, the balance between precision and recall varied greatly among the models.

**Figure 6:**
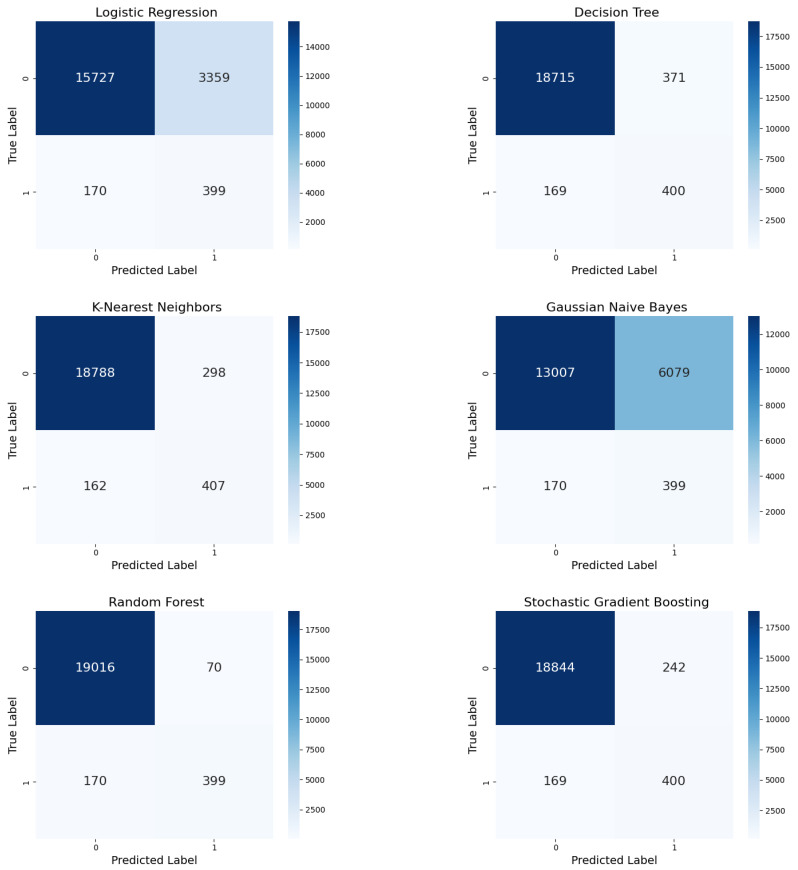
Confusion matrices for the six ML models with ADASYN oversampling.

Under ADASYN oversampling (Figure 6), the confusion matrix revealed that LR could not differentiate between the negative and positive classes due to a moderate FP rate of 3359, as opposed to stable TPs (399) and FNs (170). The number of FPs in DT reduced to 371 while balancing the TPs (400) and FNs (169) and aiming for more precise and enhanced recall. KNN secured one of the lowest FP rates (298), with comparatively stable TPs (407) and a slight reduction in FNs (162). Although GNB correctly identified 399 TPs and 170 FNs, the high number of FPs (6079) significantly reduced its precision. RF maintained high precision by minimizing FPs to 70 while maintaining TPs at 399 and FNs at 170. SGB delivered 242 FPs, 400 TPs, and 169 FNs, demonstrating exceptional performance.

**Figure 7:**
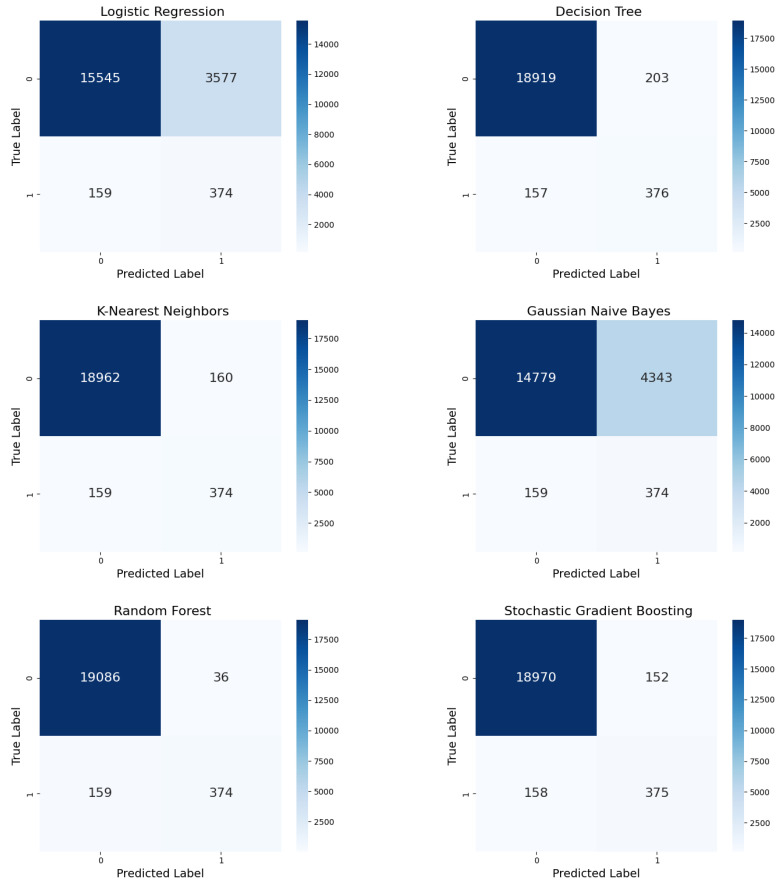
Confusion matrices for the six ML models with SMOTE oversampling.

Through SMOTE oversampling (Figure 7), LR generated more FPs (3577) than ADASYN while maintaining similar TPs (374) and FNs (159), indicating a balance between precision and recall. DT demonstrated improved performance over ADASYN in terms of reducing FPs (203), preserving high TP counts (376), and minimizing FNs (157), indicating its robustness. With fewer FPs (160), KNN performed more accurately. GNB exhibited a higher number of FPs (4343) than TPs (374) and FNs (159). RF achieved excellent results, with minimal FPs (36) and stable TPs (374) and FNs (159), demonstrating high reliability. SGB also performed well, with slightly more FPs, (152) and a maintained balance between precision and recall, with similar TPs (375) and FNs (158).

**Figure 8:**
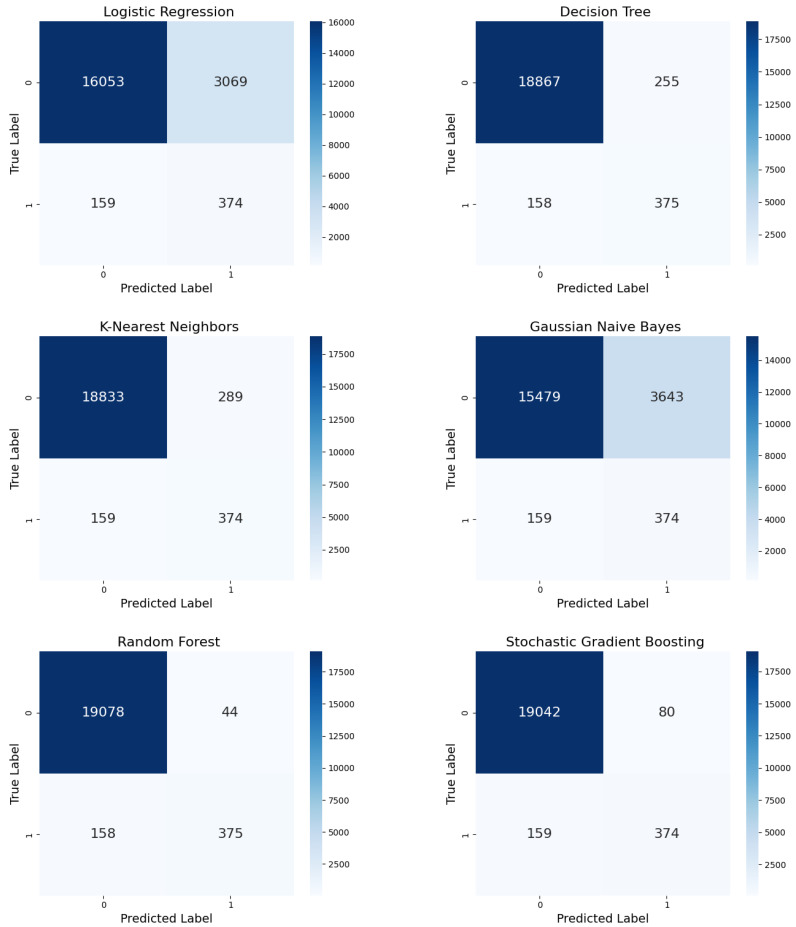
Confusion matrices for the six ML models with random oversampling.

With random oversampling, LR exhibited fewer FPs (3069) than ADASYN and SMOTE (Figure 8), with the same number of TPs (374) and FNs (159). Although DT exhibited fewer FPs (255) compared to SMOTE, it still exhibited balanced performance. KNN demonstrated robust performance, with 374 true positives (TPs) and 159 false negatives (FNs), maintaining strong precision and recall.

However, it also produced an increased number of false positives (FPs), at 289, indicating a tendency towards over-prediction of SSI cases. Under random oversampling, GNB outperformed the other Bayesian classifier, with fewer FPs (3643). RF exhibited a low error rate (44 FPs, 375 TPs, and 158 FNs). SGB reported more FPs (80) but no change in TPs (374) or FNs (159) compared to the original method.

#### 5.1.2. AUC and PR_AUC

**Figure 9:**
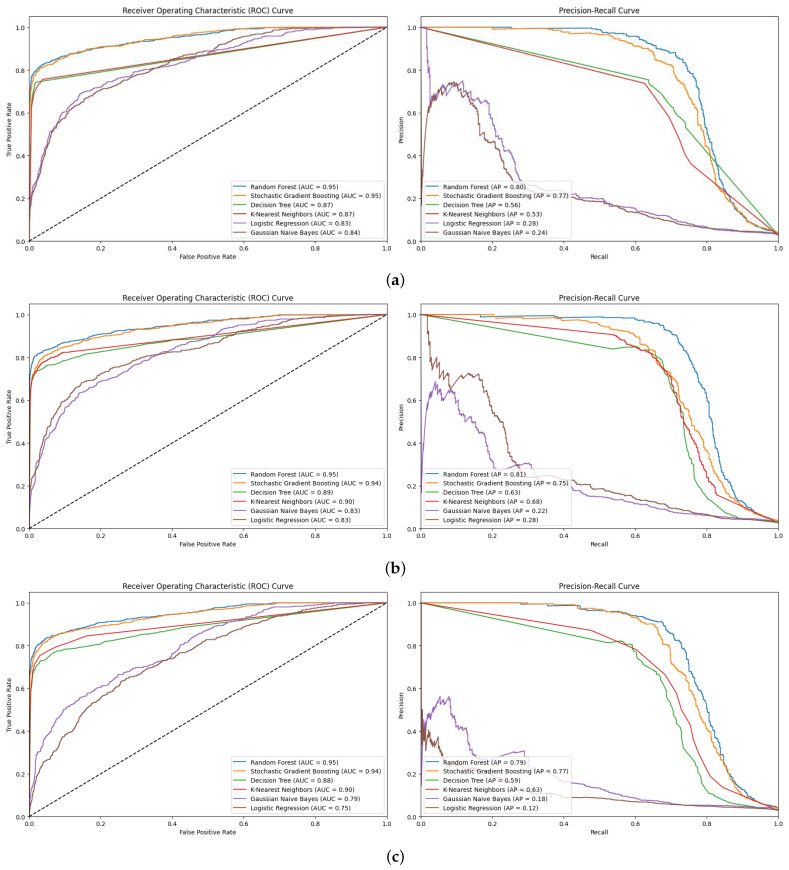
AUC and PR_AUC plots for all six ML models. (**a**) Random oversampling; (**b**) SMOTE oversampling; (**c**) ADASYN oversampling.

Figure 9 presents the AUC and PR_AUC plots for all six ML models using oversampling. RF’s consistency revealed its insensitivity to class imbalance and potential to maintain high discriminatory power. RF’s high PR_AUC values mean that high specificity and reasonably high sensitivity are essential for clinical samples [43]. Its performance on imbalanced datasets revealed exceptional results, with a maximum AUC score of 95% and PR_AUC scores between or 80%, 81%, and 79% for various oversampling techniques (Figure 9a–c). RF’s consistency and high discriminative power, which is maintained even with class imbalance, and its ability to preserve precision at higher recall levels, as indicated by elevated PR_AUC scores, are essential for clinical applications (Figure 9a–c).

SGB and RF had similar AUC scores (94–95%); however, SGB yielded slightly lower PR_AUC scores (75–77%). In imbalanced classification tasks, employing multiple evaluation metrics is critical to accurately measure performance. KNN and DT had moderate AUC values of 87–90% but significantly lower PR_AUC scores of 53–68% and 56–63%, respectively (Figure 9a–c). Although these models excelled at overall classification, they battled precision–recall dilemmas.

Across both metrics, the least favorable performance was observed for LR and GNB. These models struggled more with imbalanced data, as evidenced by a larger decrease (12–28%) in their PR_AUC scores compared to their AUC values (75–84%). The PR_AUC metric revealed a slight superiority of SMOTE over random oversampling and ADASYN when RF was used (81%).

Random oversampling outperformed SMOTE and ADASYN in the SGB and LR models. The consistently high performance across both metrics in RF and SGB, irrespective of the oversampling technique, implied an inherent capability of these ensemble methods to handle class imbalance effectively. The combination of AUC and PR_AUC evaluations led to a more thorough assessment of the imbalanced SSI prediction model’s performance. The study underscored the effectiveness of ensemble methods, such as RF, in managing imbalanced datasets in healthcare analytics and highlighted the significance of carefully selecting appropriate evaluation metrics and sampling methods to handle class imbalance.

### 5.2. Approach B: Comparison of Results Using Undersampling Techniques

Three undersampling strategies were employed to train seven ML models (LR, DT, KNN, GNB, SGB, RF, and SVM) on an imbalanced dataset to assess their impact on SSI prediction performance. Table 4 represents their performance metrics.

Under random undersampling, the recall rate of SGB and RF was 70%, with SGB exhibiting a higher precision (74%) and F1 score (70%). Furthermore, while DT and KNN performed moderately well, LR, SVM, and GNB struggled with precision and were highly sensitive to imbalanced data.

RF and SGB maintained high performance based on the IHT. Among all models, CC had the weakest precision and F1 scores; KNN yielded improved precision under IHT compared to DT; and SVM, GNB, and LR showed average performance. DT yielded high recall but poor precision compared to RF, KNN, and SGB.

Under random and IHT undersampling, RF and SGB exhibited consistently strong F1 scores (70%), while the remaining techniques—LR, SVM, and GNB—showed weak performance (precision < 10%).

CC-based undersampling demonstrated the poorest overall performance, with significantly decreased precision and F1 scores for all models. Random undersampling and iterative hard thresholding had the best performance among undersampling techniques, especially in combination with ensemble methods such as RF and SGB. For SSI prediction tasks, these models provided the optimal balance between precision and recall. CC underperformed in most models, implying its unsuitability for this application.

#### 5.2.1. Confusion Matrix

**Figure 10:**
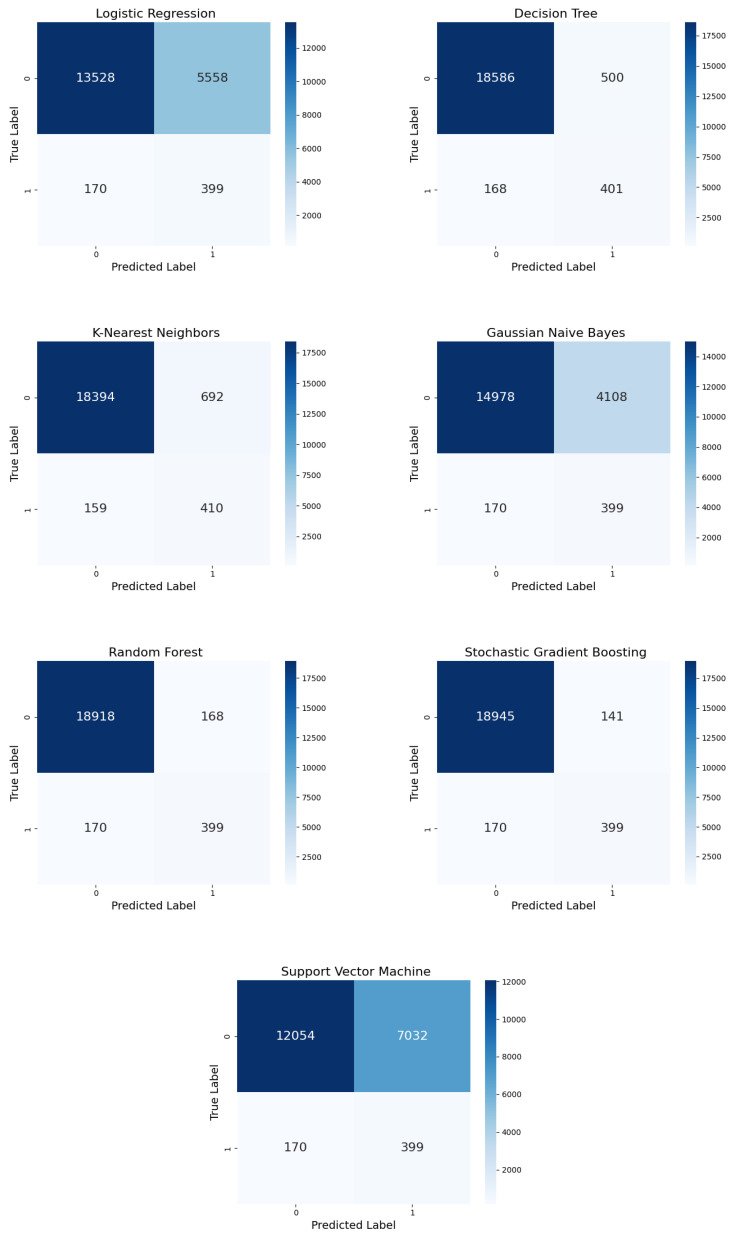
Confusion matrices for the seven ML models with random undersampling.

Figure 10 shows the comparison of the ML models with random undersampling. The performance of each model was evaluated by determining the highest number of TPs the model reported; this correctly represented the predicted positive instances. LR incorrectly classified many negative instances as positive but correctly identified 399 positive instances. This high number of FPs indicated LR’s inability to distinguish between SSI and non-SSI cases. On the other hand, DT identified a total of 438 positive cases, with significantly reduced FPs. DT outperformed LR’s sensitivity and specificity. However, KNN and DT yielded nearly identical results, with fewer FPs and 399 more TPs. KNN’s high sensitivity came with some FPs. GNB’s precision was low due to a significant number of FPs; however, it identified the same number of TPs (399) as the other models.

RF exhibited the lowest number of FPs while correctly classifying 399 positive cases. Its robustness and strong performance were evident in its ability to minimize misclassifications. SGB nearly matched RF’s performance, yielding a comparable number of FPs while identifying 399 TPs. The ensemble methods adequately dealt with imbalanced datasets because the SVM identified 474 TPs despite a notable FP count. However, the model’s enhanced sensitivity came at the expense of reduced precision.

**Figure 11:**
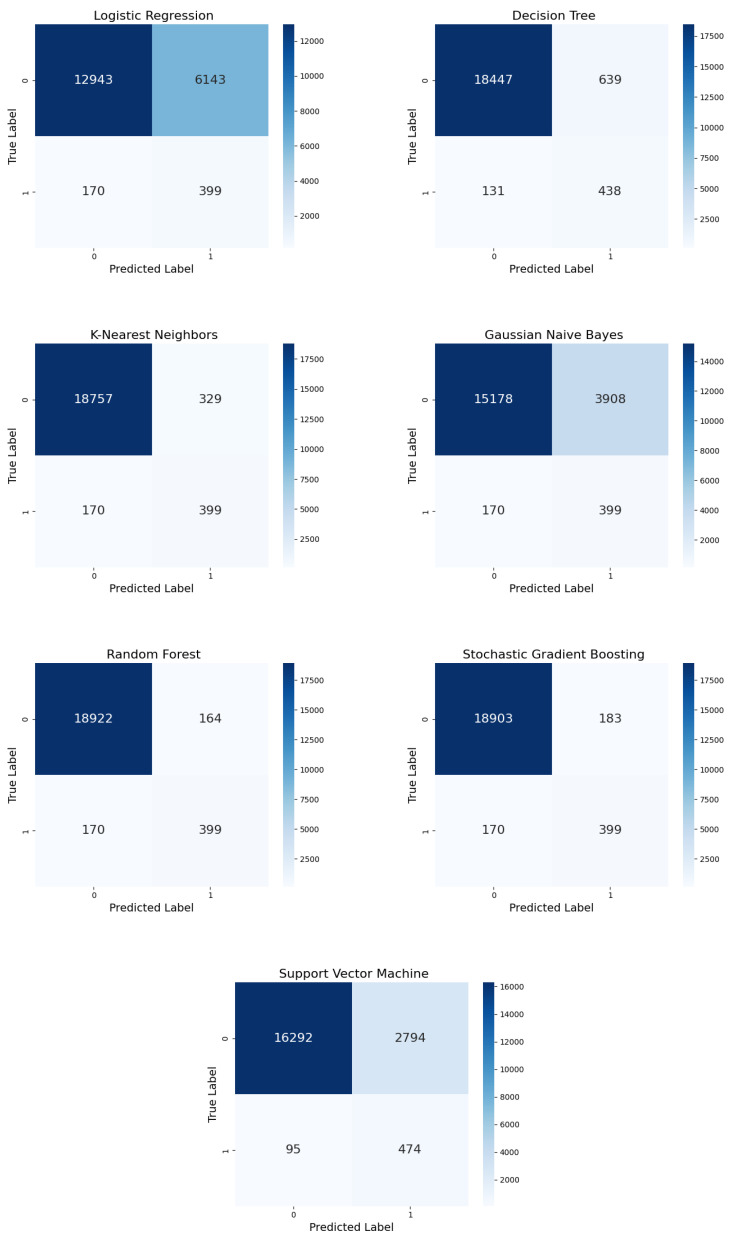
Confusion matrices for the seven ML models with IHT undersampling.

Figure 11 shows the confusion matrices for the ML models using IHT sampling. Here, 399 TPs were shared by LR and random undersampling, with a considerable number of FPs. Despite IHT, the DT model remained precise, correctly identifying 438 TPs and maintaining a low number of FPs, which is consistent with its random undersampling performance. KNN’s performance was equivalent under IHT and random undersampling, whereas GNB demonstrated a high misclassification rate, with 399 FPs. RF continued to excel, with only a few FPs and 399 TPs, demonstrating that IHT did not adversely affect its high performance. SGB also maintained a strong performance, with only a small number of FPs and 399 TPs, proving to be as robust as RF under IHT.

SVM achieved enhanced results—decreased FPs and increased TPs—when paired with IHT, although its precision is a concern. RF and SGB, as ensemble methods, showed unwaveringly high performance, irrespective of IHT sampling techniques in SSI prediction.

**Figure 12:**
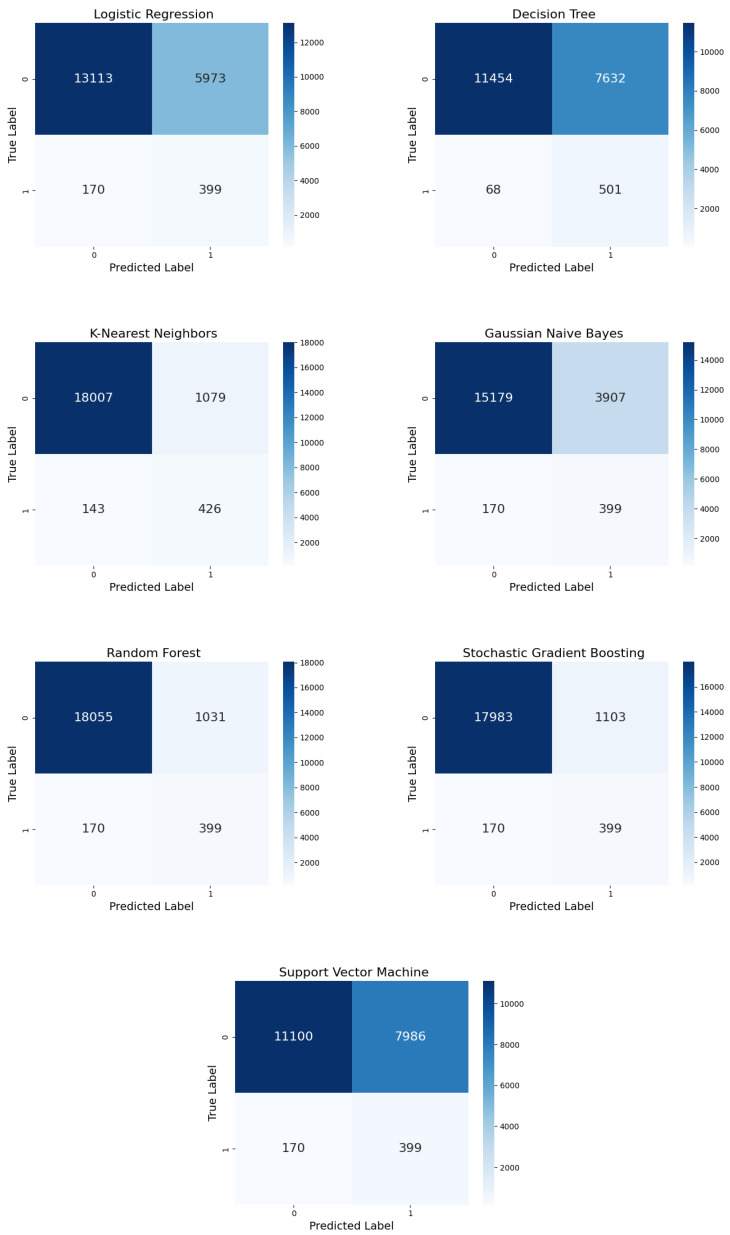
Confusion matrices for the seven ML models with CC undersampling.

Figure 12 shows the confusion matrices for the ML models with CC undersampling. The 399 TPs for LR were accompanied by a similar number of FPs, resulting in weak precision. Under CC conditions, DT misclassified numerous positive cases as negative, reducing its overall accuracy. KNN’s performance was skewed toward FPs, with a slight increase in TPs; this improved sensitivity resulted in reduced precision. Although GNB’s precision was consistent with that reported in previous studies, it remained insufficient under CC. Regarding SGB, it produced more FPs but also preserved the same number of TPs. Although it outperformed simpler models, its precision was reduced under CC. RF’s performance decreased, resulting in more FPs but the same number of TPs. Under CC, RF’s precision remained strong; however, it was negatively affected compared to random and IHT undersampling. Lastly, SVM performance was the poorest, with a substantial number of FPs.

#### 5.2.2. AUC and PR_AUC

**Figure 13:**
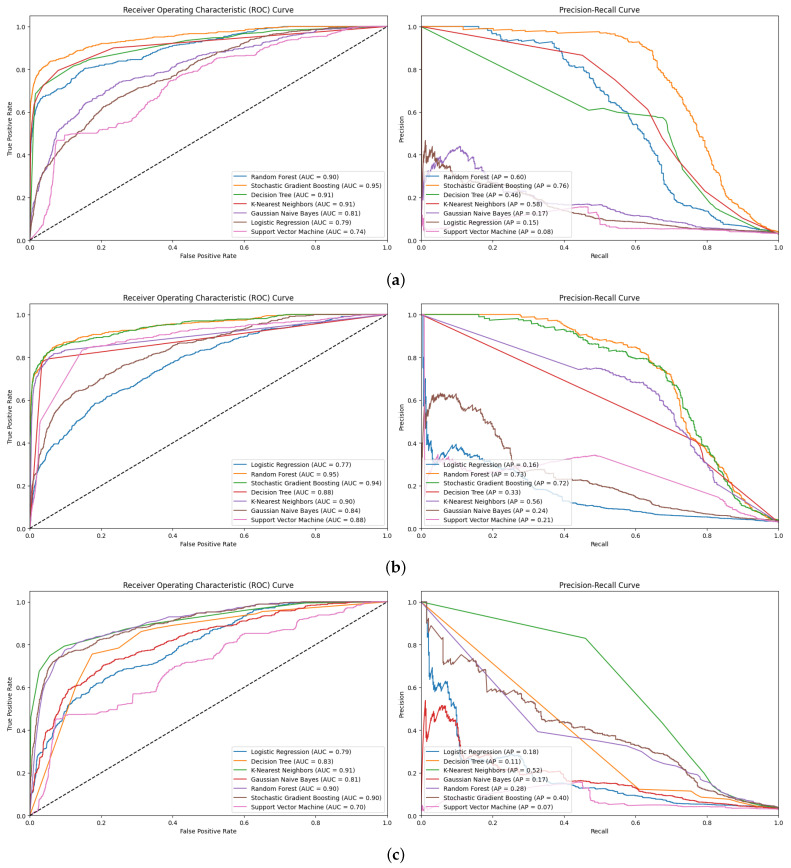
AUC and PR_AUC plots for all six ML models. (**a**) Random undersampling; (**b**) IHT undersampling; (**c**) CC undersampling.

The comparison of the AUC and PR_AUC plots significantly contributed to the analysis (Figure 13). Overall, SGB demonstrated the highest accuracy across the various undersampling methods, with random sampling resulting in the best AUC of 95% and PR_AUC of 76%. RF produced different results compared to the other three undersampling techniques. The utilization of IHT with RF resulted in AUC (95%) and PR_AUC (73%) values nearly comparable to those of SGB. Nevertheless, it significantly deteriorated with random undersampling and CC, especially in PR_AUC.

DT and KNN demonstrated moderate AUC values (88–91%) for dispersed methods; however, the PR_AUC varied precisely. KNN remained less sensitive to changes in features, as both accuracy and PR_AUC values remained nearly stable; however, for DT, the PR_AUC decreased significantly as CC increased. This signified that although both algorithms proactively retained relatively moderate levels of generality in classification ability, their recall precision at higher recoup levels was most compromised by undersampling, particularly DT with CC.

GNB, LR, and SVM yielded lower accuracy and AUC in both metrics for all undersampling metrics. The current participant sets had AUC percentages between 68% and 84% and possessed low PR_AUC scores of 7–24%. However, these results, particularly PR_AUC scores, indicated that these models failed to identify classes in the dataset.

All undersampling techniques yielded high accuracies and high AUC values of 90–95% for the top models, including SGB, RF, and KNN. The top models retained their overall classification ability. However, the greater variability in PR_AUC scores underscored the difficulty of controlling variance in such datasets, particularly when undersampling was used.

### 5.3. Comparison of Approaches A and B Using MCC Metrics

**Figure 14:**
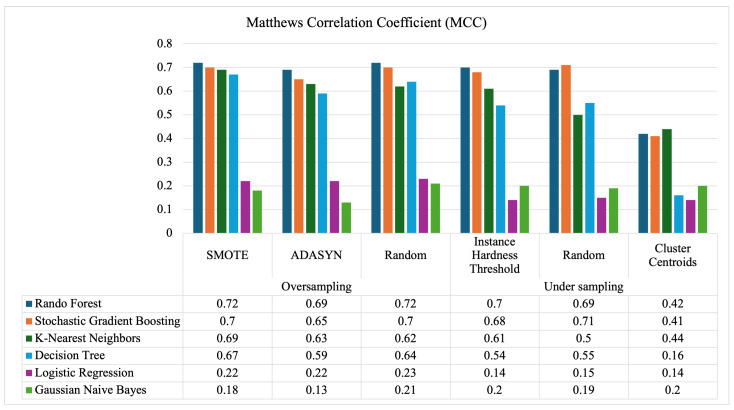
MCC results from all ML models.

Finally, the Matthews correlation coefficient (MCC) was computed, acting as an essential metric, particularly for imbalanced datasets. The current study is the first to use the MCC to evaluate an SSI prediction model. This approach is particularly beneficial for datasets with a skewed class distribution, ensuring that the performance of both the majority and minority classes is accurately represented. The MCC is valuable because it can identify models that perform well across all classes, not just the dominant one, providing a fairer comparison than metrics like accuracy or precision that can be biased toward the majority class. MCC values range from +1 to −1, where +1 indicates perfect prediction and −1 indicates complete disagreement between the predicted and actual values. Figure 14 displays the MCC values for all models, highlighting that RF achieves the highest performance, with an MCC of 0.72. The best-performing resampling technique is SMOTE, which consistently improves the performance of most ML models, except for LR and GNB. These two models perform poorly across all sampling methods. LR struggles with class imbalance due to its linear assumptions and sensitivity to skewed class distributions, which biases it toward the majority class. Similarly, GNB relies on the assumptions of feature independence and Gaussian distribution, which become unreliable when the minority class is under-represented. For computational efficiency, IHT could serve as an alternative undersampling technique, though it compromises some performance.

## 6. Discussion

ML algorithms are beneficial in identifying SSI infection and providing insights into improved clinical outcomes. LR and RF are reportedly the most suitable ML models for predicting SSIs. In addition, RF can handle sample imbalance using a bootstrap strategy, with promising results [15,44]; however, it can have low prediction or recall rates. This is because, in an unbalanced ratio of patients, the model predicts results in favor of the majority class, in which non-infected patients are detected with SSIs. Nevertheless, oversampling and undersampling techniques are potential ways to mitigate class imbalance, as explored in this research. Therefore, this study predicted the highest accuracy, recall, and precision for the RF model using undersampling and oversampling techniques.

A retrospective cohort study by Tunthanathip et al. [43] trained and tested supervised ML algorithms, such as NB, DT, ANN, and KNN, for SSI prediction. The results revealed that NB outperformed the rest, with an AUC of 76%, followed by RF, with an AUC of 0.88 (88%). RF showed good performance in predicting organ-space SSIs at the time of surgery and during oral antibiotic bowel preparation. Similarly, in the current research, RF exhibited the best performance compared to the other models, with the highest AUC of 95% when applying different oversampling or undersampling strategies. In addition, the best precision, recall, and F1 scores were achieved by combining RF with the SMOTE oversampling strategy. Da Silva et al. [44] proposed the adoption of ML methods using textual descriptions of operative records to detect SSIs; LR, RF, SBM, and SVM demonstrated SSI occurrence. In addition, the results showed that the LR model, using the feature selection technique for the oversampling and undersampling approaches, had the best predictive results (AUC 80.6%). In the current research, we observed that ML models could be misled; however, by using adequate sampling techniques and applying the feature selection strategy, the results can be enhanced.

Furthermore, SMOTE has been shown to provide significant results when used with ML classifiers for the early detection of occurrences of some serious complications, such as SSIs [11]. Substantially, in the current study, when SMOTE oversampling was adjusted with optimal hyperparameters, the RF and SGB techniques obtained the highest AUC scores (95%), with RF yielding 91% precision, surpassing the 23% precision of Xiong et al.’s [11] study, with 87% accuracy achieved using AdaBoost and SMOTE.

Due to the large dataset size, oversampling resulted in prolonged training times. We also examined alternative methods, including data reduction via undersampling and feature selection. In terms of improving both computational efficiency and prediction performance, reducing the dataset size can be advantageous.

### Limitations and Future Implications of the Study

This study has some limitations. The collected data have a retrospective nature, which means key clinical, laboratory, and microbiological details were left out. However, the data were abstracted by the staff at the participating hospitals, using strict classification definitions, ensuring accuracy and reliability. Future investigations should concentrate on prospective, randomized trials that include clinical, microbiological, and laboratory variables to determine more precise correlations and enhance prevention strategies. Healthcare data are often characterized by numerous missing values, thereby impacting model training processes and the accuracy of predictions. A large dataset can be both a benefit and a drawback, particularly regarding the variability of the information collected from multiple hospitals. Future research could focus on developing methods to consider and capitalize on this heterogeneity. Despite these limitations, we believed that this dataset was the most suitable option for testing our hypothesis. Future research should investigate various combinations of resampling techniques and incorporate approaches such as cost-sensitive learning, ensemble-based methods, or generative synthesis networks to improve generality and the robustness of classification in imbalanced data.

## 7. Conclusions

This study highlights the importance of employing oversampling and undersampling techniques alongside other preprocessing methods, such as feature selection and hyperparameter tuning, to effectively manage highly imbalanced datasets. The selection of a resampling method should be guided by the dataset’s unique description and the specific objectives of the ML model. In this research, various resampling techniques were evaluated for SSI prediction, as the dataset exhibited poor predictive performance for minority classes without such interventions. A comprehensive evaluation of seven ML models across different resampling techniques provided a holistic view of SSI prediction performance. Grid search cross-validation was applied to optimize hyperparameters and improve AUC, recall, and precision. In addition, using the MCC in this study ensured a more reliable and robust evaluation of the models’ predictive performance, especially when faced with an imbalanced dataset, where other metrics may fail to provide an accurate picture of model performance.This work contributes to the literature by advancing strategies for handling imbalanced classification in real-world scenarios.

## Figures and Tables

**Table 1:** Summary of related work.

Reference	Class Distribution		Classifier Models	Balancing Method	Feature Selection	Performance
[15]	SSI-cases	542 (3.2%)	LR and RF	No	Previous literature and clinical experts	AUC: 68%
	Total	16,842				
[16]	SSI-cases	205 (2.2%)	LR, DT, SVM	No	No	AUC: 73%
	Total	9274				
[17]	SSI-cases	2751 (10%)	LR, GB, ANN	No	No	AUC: 76%
	Total	275,152				
[7]	SSI-cases	9460 (0.32%)	LR, RF, DT, SVM, ANN, DNN	No	No	AUC: 85%
	Total	2,882,526				
[10]	SSI-cases	795 (5.5%)	RF	No	Stepwise feature	AUC: 89%
	Total	14,351			selection	
[11]	SSI-cases	33 (5.65%)	ADA-Boost, LR XGBoost	SMOTE	No	AUC: 87%
	Total	584	Stochastic (GB)			

**Table 2:** Hyperparameters of the models.

Sampling Technique and Its Strategy	ML Model	Hyperparameters
SMOTE {sampling_strategy = 0.2, k_neighbors = 7}	LR	C = 10, penalty = ‘12’, solver = ‘newton-cg’.
	DT	Max_depth = 15, min_samples_leaf = 4, ‘min_samples_split’ = 10.
	KNN	Algorithm = ‘auto’, n_neighbors = 11, weights = ‘distance’.
	GNB	Var_smoothing = 1 × 10^−9^.
	RF	Max_depth = None, min_samples_leaf = 2, min_samples_split = 2, n_estimators = 200.
	SGB	Max_iter = 200, learning_rate = 0.01, max_leaf_nodes = 31, random_state = 42)
ADASYN {sampling_strategy = 0.5, random_state = 42}.	LR	C = 10, penalty = ‘12’, solver = ‘newton-cg’.
	DT	Max_depth = 15, min_samples_leaf = 4, ‘min_samples_split’ = 10.
	KNN	Algorithm = ‘kd_tree’, n_neighbors = 11, weights = ‘distance’.
	GNB	Var_smoothing = 1 × 10^−9^.
	RF	Max_depth = None, min_samples_leaf = 2, min_samples_split = 2, n_estimators = 200.
	SGB	Max_iter = 200, learning_rate = 0.01, max_leaf_nodes = 31, random_state = 42)
Random over Sampling {sampling_strategy = 0.2, random_state = 42}	LR	C = 100, penalty = ‘12’, solver = ‘newton-cg’.
	DT	Max_depth = None, min_samples_split = 5, min_samples_leaf = 4.
	KNN	Algorithm = ‘auto’, n_neighbors = 5, weights = ‘distance’.
	GNB	Var_smoothing = 1 × 10^−9^.
	RF	Max_depth = None, min_samples_leaf = 2, min_samples_split = 10, n_estimators = 300.
	SGB	Max_iter = 200, learning_rate = 0.01, max_leaf_nodes = 31, random_state = 42)
Random Undersampling {sampling_strategy = 0.2}	LR	C=10, penalty = ‘12’, solver = ‘newton-cg’.
	DT	Max_depth = 10, min_samples_leaf = 4, ‘min_samples_split’ = 10.
	KNN	Algorithm = ‘auto’, n_neighbors = 11, weights = ‘uniform’.
	GNB	Var_smoothing = 1 × 10^−8^.
	RF	Max_depth = None, min_samples_leaf = 1, min_samples_split = 2, n_estimators = 200.
	SGB	Max_iter =200, learning_rate =0.01, max_leaf_nodes =31, random_state =42)
	SVM	Class_weight = ‘balanced’, C = 0.1, gamma = 0.1, kernel = ‘rbf’, probability = True.
Cluster Centroid {sampling_strategy = 0.5}	LR	C = 10, penalty = ‘12’, solver = ‘newton-cg’, class_weight = ‘Balanced’.
	DT	Max_depth =10, min_samples_split = 5, min_samples_leaf =1.
	KNN	Algorithm = ‘auto’, n_neighbors =11, weights = ‘uniform’.
	GNB	Var_smoothing = 1 × 10^−8^
	RF	Max_depth = None, min_samples_split =10, random_state =2, min_samples _leaf =1, n estimators =200.
	SGB	Max_iter =200, learning_rate =0.01, max_leaf_nodes =31, random_state =42)
	SVM	class_weight = ‘balanced’, probability = True.
Instance hardness thresholds (IHT). {estimator = Random Forest Classifier (random_state = 42)}	LR	Class_weight = ‘balanced’.
	DT	max_depth =1000, min_samples_split = 5, random_state =42.
	KNN	N_neighbors = 11, algorithm = ‘auto’, weights = ‘distance’.
	GNB	Default
	RF	Max_depth = None, min_samples_leaf = 2, min_samples_split = 5, random_state = 42.
	SGB	Max_iter =200, learning_rate =0.01, max_leaf_nodes =31, random_state =42)
	SVM	Class_weight = ‘balanced’, C = 0.1, gamma = 0.1, kernel = ‘rbf’, probability = True

**Table 3:** Performance metrics of ML algorithms under approach A.

ML	Oversampling	Acc %	Precision % (1)	Recall % (1)	F1 Score % (1)	AUC %	PR_AUC %
RF	SMOTE	99	91	70	79	95	81
RF	Random	99	89	70	79	95	80
RF	ADASYN	99	85	70	77	95	79
SGB	Random	99	82	70	76	95	77
SGB	SMOTE	99	71	70	71	94	75
KNN	SMOTE	98	70	70	70	90	68
DT	SMOTE	98	65	71	68	89	63
SGB	ADASYN	98	62	70	66	94	77
DT	Random	98	60	70	64	87	56
KNN	ADASYN	98	58	72	64	90	63
KNN	Random	98	56	70	63	87	53
DT	ADASYN	97	52	70	60	88	59
LR	Random	84	11	70	19	83	28
LR	ADASYN	82	11	70	18	75	12
LR	SMOTE	81	9	70	17	83	28
GNB	Random	81	9	70	16	84	24
GNB	SMOTE	77	8	70	14	83	22
GNB	ADASYN	68	6	70	11	79	18

**Table 4:** Performance metrics of ML algorithms using approach B.

ML	Undersampling	Acc %	Precision % (1)	Recall % (1)	F1 Score % (1)	AUC %	PR_AUC %
SGB	Random	98	74	70	70	95	76
RF	IHT	98	71	70	70	95	73
SGB	IHT	98	69	70	69	94	72
RF	Random	98	70	70	70	90	60
KNN	Random	96	37	72	49	91	58
KNN	IHT	97	55	70	62	90	56
KNN	CC	94	28	75	41	91	52
DT	Random	97	45	70	55	91	46
SGB	CC	94	27	70	39	90	40
DT	IHT	96	41	77	53	88	33
RF	CC	94	28	70	40	90	28
GNB	IHT	79	9	70	16	84	24
SVM	IHT	83	15	82	25	88	21
LR	CC	69	6	70	11	79	18
GNB	Random	78	9	70	16	81	17
GNB	CC	79	9	70	16	81	17
LR	IHT	68	6	70	11	77	16
LR	Random	71	7	70	12	79	15
DT	CC	61	6	88	12	83	11
SVM	Random	63	5	70	10	68	8
SVM	CC	59	5	70	9	70	7

## Data Availability

The data that support the findings of this study are not publicly available due to privacy and ethical considerations.

## Appendix A

**Table A1:** The baseline characteristics of study participants based on the presence of an SSI event.

	Presence of SSI (Mean ± SD)	*p*-Value		
	Total	NO	YES	
**Weight (kg)**	76.3 ± 14.1	76.2 ± 14.0	77.7 ± 15.5	0.000
**Procedure duration (minutes)**	49.8 ± 26.9	49.6 ± 26.7	60.9 ± 37.5	0.000
**Height (cm)**	158.10 ± 5.9	158.0 ± 5.9	158.7 ± 5.9	0.000
**Core Temperature (°C)**	37.5 ± 0.9	37.5 ± 1.0	37.6 ± 0.9	0.000
**BMI**	30.62 ± 5.9	30.61 ± 5.9	30.9 ± 6.5	0.000

This table presents a detailed analysis of the numerical variables of individuals with an without an SSI. Data are represented as mean and Standard Deviation (SD). Based on results of the Mann–Whitney U-test, for all numerical variables, *p*-values less than 0.05 were considered statistically significant. BMI: Body Mass Index.

**Table A2:** The baseline clinical characteristics of study participants by occurrence of surgical site infections (SSIs).

Factor		SSI_EVENT N (%)		Total = 64,793	*p* Value
		NO Count = 63,161	YES Count = 1632		
ASA SCORE	I	35,917 (97.9)	781 (2.1)	36,698	0.000
	II	24,815 (97.8)	569 (2.2)	25,384	
	III	1928 (91.2)	186 (8.8)	2114	
	IV	383 (83.8)	74 (16.2)	457	
	V	118 (84.3)	22 (15.7)	140	
Temperature	No	4875 (98.9)	55 (1.12)	4930	0.000
Normality	Yes	62,155 (97.5)	1577 (2.5)	63,732	
Serum Glucose	NO	12,365 (97.7)	287 (2.3)	12,652	0.000
	YES	15,034 (96.7)	516 (3.3)	15,550	
Day 1 Level	NA	35,762 (97.7)	829 (2.3)	36,591	
Serum Glucose	NO	12,774 (97.6)	312 (2.4)	13,086	0.000
	YES	13,092 (96.5)	471 (3.5)	13,563	
Day 2 Level	NA	37,259 (97.7)	849 (2.3)	38,144	
Trauma	NO	60,788 (97.8)	1347 (2.2)	62,135	0.000
	YES	2373 (89.3)	285 (10.7)	2658	

This table presents the distribution of various clinical characteristics of categorical variables by the occurrence of surgical site infections (SSIs). Data are represented as frequency (N) and percentage (%). The table also includes *p*-values indicating the statistical significance of the associations between the factors and SSI occurrence. ASA Score: American Society of Anesthesiologists Score.

**Table A3:** The baseline surgical procedure characteristics of study participants by occurrence of surgical site infections (SSIs).

Factor		SSI_EVENT N (%)		Total = 64,793	*p* Value
		NO Count = 63,161	YES Count = 1632		
Admission Type	Elective	24,774 (97.7)	577 (2.3)	25,351	0.0017
	Emergency	38,387 (97.3)	1055 (2.7)	39,442	
Overall Compliance	NO	30,272 (97.3)	829 (2.7)	31,101	0.0235
	YES	32,889 (97.6)	803 (2.4)	33,692	
Hair Clipped	NO	9925 (96.7)	344 (3.3)	10,269	0.000
	NA	11,510 (97.0)	361 (3.0)	11,871	
	YES	41,726 (97.8)	927 (2.2)	42,653	
Antibiotic before Surgery	NO	11,066 (96.5)	402 (3.5)	11,469	0.000
	YES	52,095 (97.7)	1229 (2.3)	53,324	
Prophylactic Antibiotic	NO	12,204 (96.4)	451 (3.6)	12,655	0.000
	YES	50,957 (97.7)	1181 (2.3)	52,138	
Discontinuation of Prophylactic	NO	20,045 (96.8)	662 (3.2)	20,707	0.000
	YES	43,116 (97.8)	970 (2.2)	44,086	
Multiple Procedures	NO	62,178 (97.6)	1520 (2.4)	63,698	0.000
	YES	983 (89.8)	112 (10.2)	1095	
Laparoscope/Endoscope	NO	57,317 (97.4)	1516 (2.6)	58,833	0.0035
	YES	5844 (98.1)	116 (1.9)	5960	
Closure Technique	Primary	62,521 (97.5)	1607 (2.5)	64,128	0.000
	Other primary	640 (96.2)	25 (3.8)	665	
Wound Class	Clean	32,059 (97.7)	756 (2.3)	32,815	0.000
	Clean-Contaminated	29,873 (97.8)	663 (2.2)	30,536	
	Contaminated	1003 (88.6)	129 (11.4)	1132	
	Dirty	226 (72.9)	84 (27.1)	310	
General Anesthesia	NO	33,232 (98.3)	590 (1.7)	33,822	0.000
	YES	29,929 (96.6)	1042 (3.4)	30,971	
Procedure Name	Cesarean Section (CSEC)	49,838 (98.4)	818 (1.6)	50,656	0.000
	Gallbladder surgery (CHOL)	4273 (99.2)	34 (0.8)	4307	
	Appendix surgery (APPY)	3629 (97.2)	105 (2.8)	3734	
	Open reduction of fracture (FX)	3244 (93.8)	214 (6.2)	3458	
	Exploratory laparotomy (XLAP)	497 (70.6)	207 (29.4)	704	
	Gastric surgery (GAST)	537 (98.0)	11 (2.0)	548	
	Herniorrhaphy (HER)	255 (92.7)	20 (7.3)	275	
	Thyroid and/or parathyroid surgery (THYR)	74 (98.7)	1 (1.3)	75	
	Cardiac surgery (CARD, CBGB, and CBCG)	420 (86.6)	65 (13.4)	485	
	Amputation (AMP)	113 (75.8)	36 (24.2)	149	
	Craniotomy (CRAN)	100 (74.1)	35 (25.9)	135	
	Others (Ventricular shunt VSHN, Spleen surgery SPLE, Small bowel surgery SB, Rectal surgery REC, Prostate surgery PRST, Ovarian surgery OVRY, Kidney surgery NEPH, Neck surgery NECK, Laminectomy LAM, Abdominal hysterectomy HYST, Spinal fusion FUSN, Colon surgery COLO, Breast surgery BRST, Shunt for dialysis AVSD)	195 (70.9)	80 (29.1)	275	
Surveillance period	Thirty	58,743 (97.9)	1247 (2.1)	59,990	0.000
	Ninety	4418 (92.0)	385 (8.0)	4803	
Location	Yes	3800 (98.67)	51 (1.32)	3851	0.0000
	No	59,361 (97.40)	1581 (2.59)	60,942	
Pre-procedure diagnosis	* PRE_PROCEDURE_DIAGNOSIS_GROUPED				0.000

This table summarizes various procedure-related characteristics of categorical variables and their association with the occurrence of surgical site infections (SSIs). Data are presented as frequency (N) and percentage (%). The table also includes *p*-values indicating the statistical significance of the associations between the factors and SSI occurrence.

* We addressed variables with a high number of categories by consolidating them into fewer, more meaningful bins. This grouping process simplified the data, improved the accuracy of statistical tests, and retained essential information.
